# Supplementary material for: Genome-scale reconstructions of the mammalian secretory pathway predict metabolic costs and limitations of protein secretion
Source: Nat Commun. 2020 Jan 2;11:68. doi: 10.1038/s41467-019-13867-y (PMC6940358; doi:10.1038/s41467-019-13867-y)
Supplement: Supplementary file 1 — Supplementary Information [file 41467_2019_13867_MOESM1_ESM.pdf]

Supplementary Information for

"Genome-scale reconstructions of the mammalian secretory pathway predict metabolic costs and limitations of protein secretion"

Gutierrez et al.

Contents

- Supplementary Discussion
- Supplementary Figure 1
- Supplementary Figure 2
- Supplementary Figure 3
- Supplementary Table 1
- Supplementary Table 2
- Supplementary References

Supplementary Discussion

Overview of the Secretory Pathway in animal cells

Historically, most of the knowledge on the secretory pathway was obtained by studying protein transport processes and secretion in *Saccharomyces cerevisiae*[1]. Albeit quite similar in core functions, the secretory pathways of mammalian cells and fungi differ significantly in some of the steps which have been evolved based on species-specific secretion phenotypes[2]. The following paragraphs briefly overview the mammalian secretory pathway and highlights pathways exclusive to animals not present in fungi. The last section provides an in-depth comparison of the yeast and animal secretory pathways while highlighting the most important differences between both.

Translocation and processing in endoplasmic reticulum

Proteins destined to the secretory pathway generally bear a signal peptide at the amino-terminus which targets the proteins to the endoplasmic reticulum (ER) where the initial post-translational modifications (PTMs) take place. This transport requires translocating the target protein across the ER membrane through two general pathways: co-translational translocation (GTP dependent) and post-translational translocation (ATP dependent)[3]. An additional pathway for tail-anchored (TA) proteins into the ER membrane has also been discussed in the literature and included in our iCHO1921s reconstruction[4,5]. Once inside the ER lumen, the target proteins are folded by the action of several transmembrane ER proteins, including calnexin, calreticulin, and other luminal chaperones[6–8]. In the event of protein misfolding, a target protein may go through a “quality control” system (exclusive in the mammalian secretory pathway) that attempts to correct for folding errors[9,10]. However, if the misfolded state of the protein is sustained for too long, the protein then enters the ER associated degradation pathway, or ERAD, which involves retrotranslocation of the misfolded protein back to the cytosol, ubiquitination and proteasomal degradation[11–13].

Besides folding, a target protein may acquire additional PTMs while inside the ER such as attachment of a glycosylphosphatidylinositol (GPI) anchor[14,15], formation of disulfide bonds[16], and N-linked glycosylation[17–20]. After these PTMs are successfully completed , the target

proteins are transported to the Golgi apparatus via COPII-coated vesicles that bud from the ER[21,22] whereas misfolded proteins are retro-translocated to the cytoplasm[23,24] for proteasomal degradation via the ER-associated degradation pathway (ERAD)[25,26]. In the Golgi apparatus, N-glycans are processed into branched and complex glycoforms and proteins are further glycosylated with O-linked glycans[27–29] and then sorted to their final destination (e.g. lysosome, extracellular medium) via clathrin-coated secretory vesicles[30–33].

A note on translocation pathways

In co-translational translocation, proteins destined to the secretory pathway bear a hydrophobic signal sequence at the amino-terminus that promotes the targeting of ribosome-nascent chain (RNC) complexes to the ER via binding to the signal recognition particle (SRP). The SRP recognizes the signal peptide as soon as it emerges from the ribosome during translation. Then, the newly formed SRP-RNC complex is recognized by the SRP receptor on the ER membrane where translocation is initiated by interaction with the Sec61 complex (Sec61C) and assisted by the chaperone BiP to increase the efficiency and ensure the unidirectionality of this process[30].

Post-translational translocation, in contrast to co-translational translocation, occurs independently of SRP and its receptor[34]. Furthermore, this process does not rely too heavily on the Sec61C to translocate the target protein and instead utilizes the protein Sec62 as a safe route that guarantees the efficient translocation of small proteins (<160 amino acids in length)[35].

Finally, the pathway for inserting TA proteins into the ER membrane also occurs post-translationally due to the fact that the ER targeting signal of TA proteins is located very close to the carboxy-terminus, which allows the ribosome to release the protein before it is recognized and localized to the ER[36]. This pathway depends on ATP and one of the main players in the process is a transmembrane recognition complex known as TRC40 or Asna1[37].

Important differences between the yeast and animal secretory pathways

As mentioned above, core functions of the secretory pathway are conserved between mammalian and yeast cells. These core functions (see Supplementary Table 2) are:

- Translocation through endoplasmic reticulum
- Primary glycosylation in ER (N-linked glycans) and Golgi (N-linked and O-linked glycans)
- Protein folding and quality control in ER
- Anterograde and retrograde vesicular transport between ER and Golgi via COPII and COPI vesicles, respectively.
- Dolichol pathway for N-linked core glycan translocation through the ER membrane
- Endoplasmic reticulum associated degradation (ERAD)
- GPI biosynthesis
- Unfolded protein response (UPR)

Nevertheless, minor and major differences exist between the yeast and mammalian secretory pathways. Some of these differences have been thoroughly reviewed before in an excellent review by Delic and colleagues[2] and are summarized in Supplementary Table 1, which highlights the major differences between both secretory pathways that are relevant for modeling purposes using the secretory reconstructions. Finally, Supplementary Table 2 summarizes the differences between the mammalian and the fungal secretory pathway reconstructions in terms of components, reactions, and subsystems.

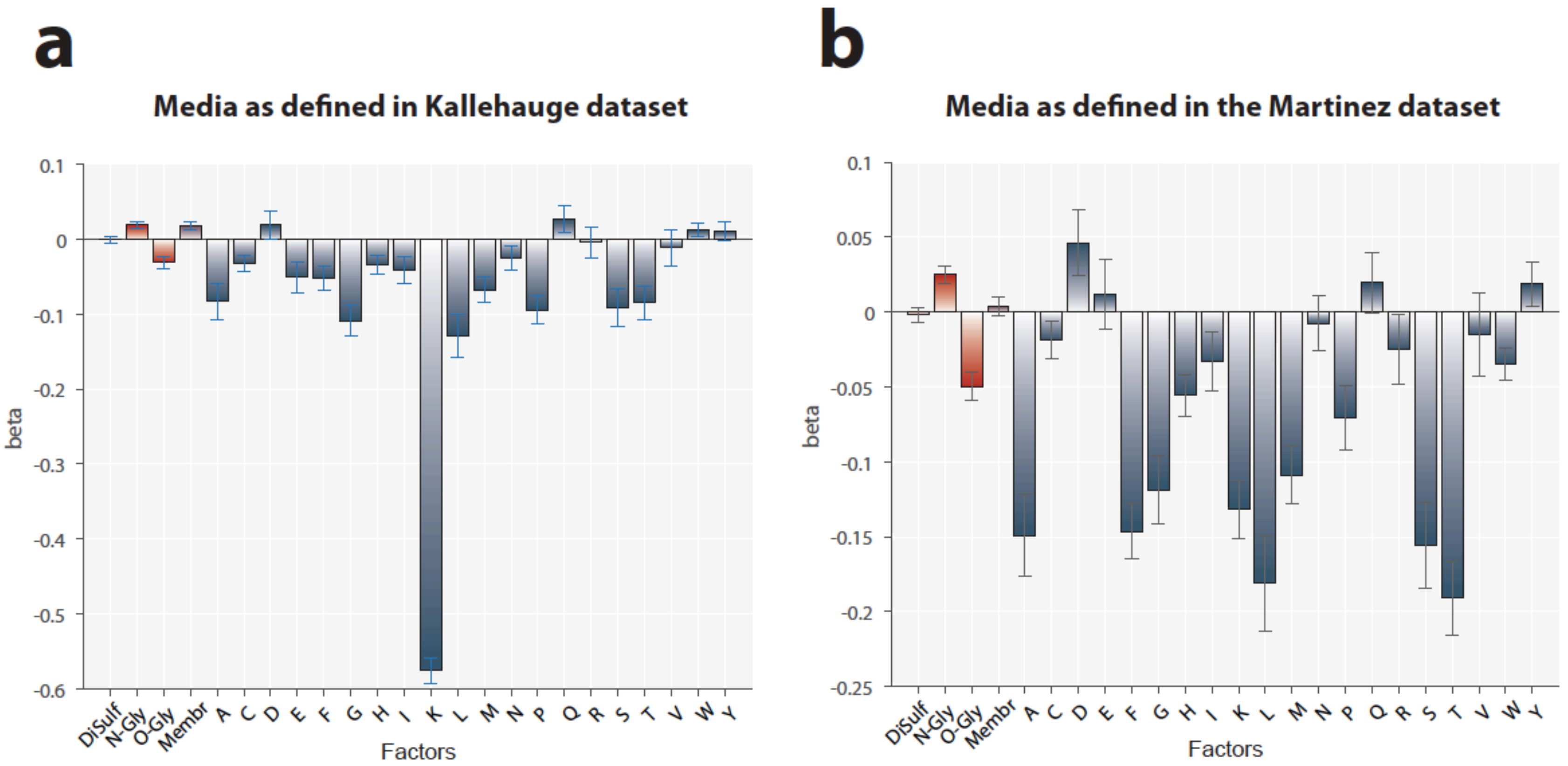

Supplementary Figure 1 – Factors affecting iCHO2048s-predicted productivity with two different media compositions. Linear regression coefficients (β) to quantify the contribution of PTMs to the explained variation in specific productivity using uptake rates different from those used in Figure 4c. The specific consumption rates are listed in Supplementary Table 3 as Kallehauge[38] (left panel) and Martinez[39] (right panel). Error bars represent the standard errors of the fitted coefficients. Source data are provided as a Source Data file.

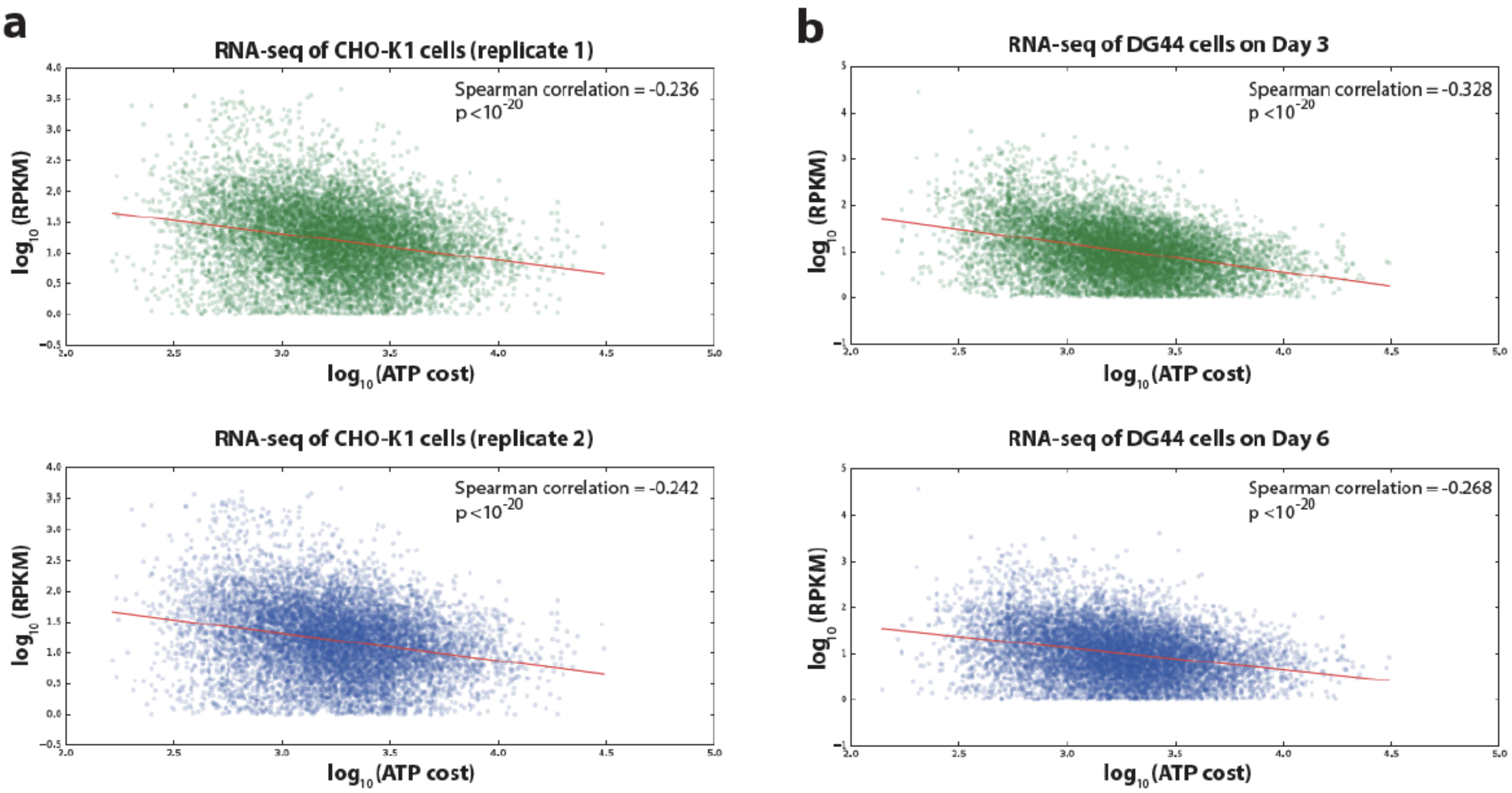

Supplementary Figure 2 – Spearman correlation between ATP cost and RNA-seq gene expression levels. RNA-seq gene transcription levels from (a) a non-producing CHO-K1 cell line[40] and (b) a mAb-producing CHO-DG44 cell line[38] were compared against the ATP cost of producing the translated proteins. Source data are available as a Source Data file.

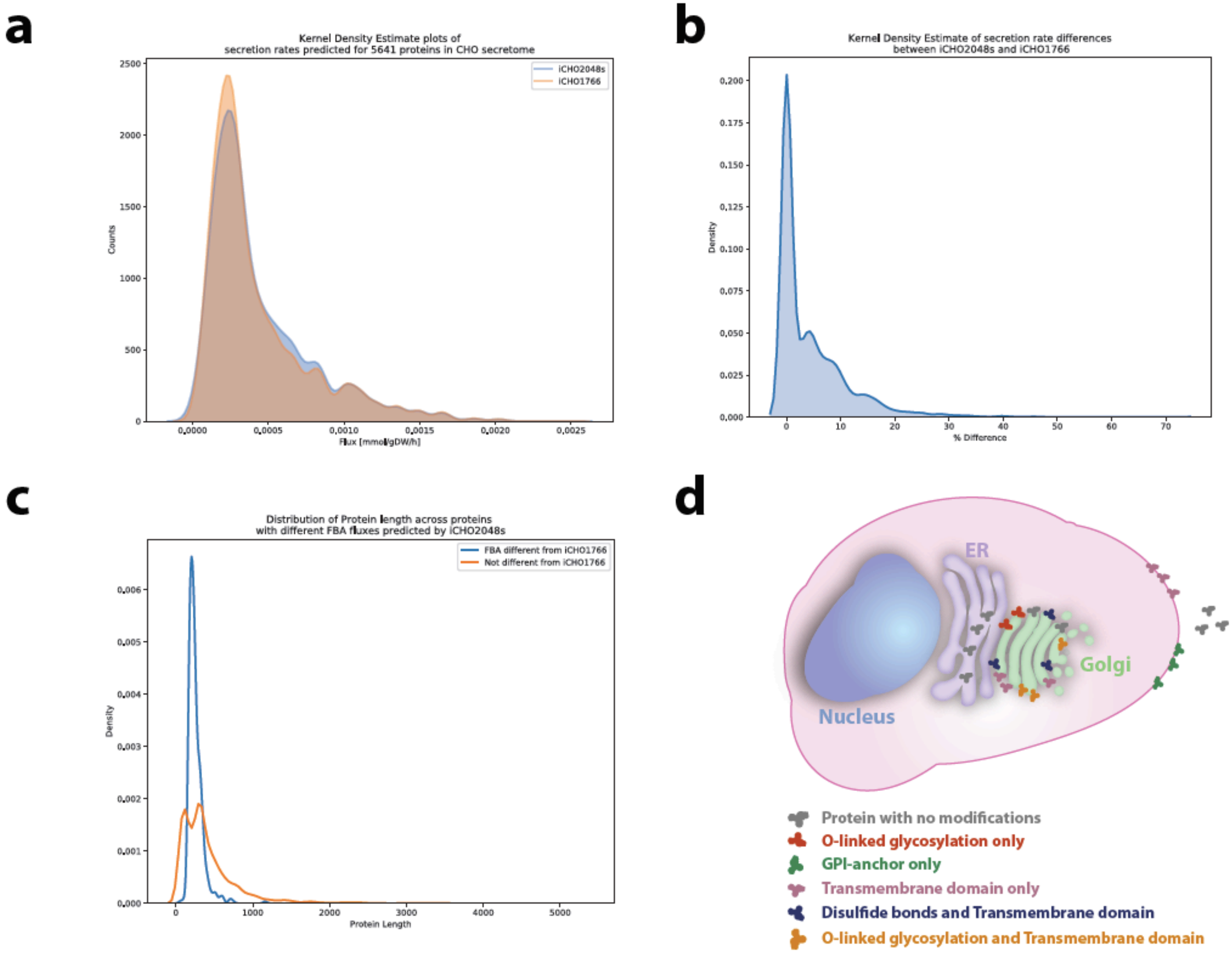
a
Kernel Density Estimate plots of
secretion rates predicted for 5641 proteins in CHO secretome
iCHO2048s
iCHO1766
Counts
Flux [mmol/gDW/h]
b
Kernel Density Estimate of secretion rate differences
between iCHO2048s and iCHO1766
Density
% Difference
c
Distribution of Protein length across proteins
with different FBA fluxes predicted by iCHO2048s
FBA different from iCHO1766
Not different from iCHO1766
Density
Protein Length
d
ER
Golgi
Nucleus
Protein with no modifications
O-linked glycosylation only
GPI-anchor only
Transmembrane domain only
Disulfide bonds and Transmembrane domain
O-linked glycosylation and Transmembrane domain

Supplementary Figure 3 – Comparison of secretion rates predicted by iCHO2048s and iCHO1766. Kernel Density Plots of (a) secretion rates for 5641 proteins in the CHO secretome, as computed with iCHO2048s (blue) and iCHO1766 (orange), (b) the percentage difference between predictions with iCHO2048s and iCHO1766, and (c) the protein lengths (amino acids in sequence) of proteins showing a secretion rate difference in both models (blue) or not (orange). iCHO2048s predicts different fluxes for proteins with a specific posttranslational modification profile, size, and localization. For about 8% of the target secretome, secretion rates predicted with iCHO2048s are at least 15% different from their iCHO1766 counterparts. Interestingly, this 8% corresponds to short (less than 350 amino acids) secreted proteins with O-linked glycans, GPI anchors or transmembrane domains whose final location is the extracellular space, the ER lumen, the Golgi membrane, or the plasma membrane, as summarized in (d). Thus, for a proportion of the secretome, there are non-negligible energetic and synthetic costs associated with vesicular transport, protein folding, and membrane anchoring only accounted for when iCHO2048s couples to metabolism. A detailed description of the results, as well as the source data, can be found in Jupyter Notebook E at https://github.com/LewisLabUCSD/MammalianSecretoryRecon/JUPYTER_NOTEBOOKS

Supplementary Table 1 – Summary of differences between mammalian and yeast secretory pathways as described by Delic et al.[2]

Description of difference	Mammalian secretory pathway	Yeast secretory pathway	Importance for modeling purposes
Chaperones involved in translocation	The main chaperone is BiP	The main chaperone is Kar2	Minor
Presence of heat-shock proteins (HSPs) in ER	Mainly presence of proteins in the Hsp90 family	Not found in yeast	Minor
Enzymes for detoxification of reactive oxygen species in ER	Contains several enzymes such as Ero1 and glutathione peroxidases	Not found in yeast	Major
Oxidation state of Protein disulfide isomerase (PDI)	PDI is mainly reduced	PDI is mainly oxidized	Minor
Components of calnexin-calreticulin cycle	Includes an enzyme coded by the UGGT gene to transfer glucose residues to core N-linked glycans in misfolded proteins	Lacks UGGT and instead directs misfolded proteins to ER exit	Major
ERAD pathway branches for degrading misfolded proteins	Capable of directing misfolded proteins towards the ERAD pathway by trimming N-linked glycan residues in the A, B and C branches	Capable of directing misfolded proteins towards the ERAD pathway by trimming N-linked glycan residues only in B and C branches	Major
Components of COPII vesicles	Contains four isoforms of Sec24	Expresses Sec24 with three cargo binding sites as well as Sec24 homologs Sfb2-3	Minor

Supplementary Table 2 – Main differences between the mammalian and yeast secretory pathway reconstructions

<table>
<tr><th>Secretory pathway reconstruction</th><th>Number of components</th><th>Number of reactions</th><th>Number of Subsystems</th><th>Core subsystems (in both mammalian and yeast secretory pathways)</th><th>Unique subsystems</th></tr>
<tr><td>Mammalian</td><td>271</td><td>144</td><td>12</td><td rowspan="2">A total of 9 core subsystems: COPI, COPII, Dolichol pathway, ER glycosylation, ERAD, Golgi processing, GPI biosynthesis, Protein folding, and Translocation</td><td>Clathrin vesicles, GPI transfer</td></tr>
<tr><td>Yeast</td><td>165</td><td>137</td><td>16</td><td>ALP pathway, CPY pathway</td></tr>
</table>

Supplementary References

1. Schekman, R. & Novick, P. 23 genes, 23 years later. Cell 116, S13–5, 1 p following S19 (2004).
2. Delic, M. et al. The secretory pathway: exploring yeast diversity. FEMS Microbiol. Rev. 37, 872–914 (2013).
3. Mandon, E. C., Trueman, S. F. & Gilmore, R. Protein translocation across the rough endoplasmic reticulum. Cold Spring Harb. Perspect. Biol. 5, (2013).
4. Shao, S. & Hegde, R. S. Membrane Protein Insertion at the Endoplasmic Reticulum. Annu. Rev. Cell Dev. Biol. 27, 25–56 (2011).
5. Borgese, N. & Fasana, E. Targeting pathways of C-tail-anchored proteins. Biochim. Biophys. Acta - Biomembr. 1808, 937–946 (2011).
6. Gidalevitz, T., Stevens, F. & Argon, Y. Orchestration of secretory protein folding by ER chaperones. Biochim. Biophys. Acta 1833, 2410–24 (2013).
7. Araki, K. & Nagata, K. Protein folding and quality control in the ER. Cold Spring Harb. Perspect. Biol. 3, a007526 (2011).
8. Braakman, I. & Hebert, D. N. Protein folding in the endoplasmic reticulum. Cold Spring Harb. Perspect. Biol. 5, a013201 (2013).
9. Sousa, M. & Parodi, A. J. The molecular basis for the recognition of misfolded glycoproteins by the UDP-Glc:glycoprotein glucosyltransferase. EMBO J. 14, 4196–203 (1995).
10. Caramelo, J. J. & Parodi, A. J. Getting In and Out from Calnexin/Calreticulin Cycles. J. Biol. Chem. 283, 10221–10225 (2008).
11. Xu, C. & Ng, D. T. W. Glycosylation-directed quality control of protein folding. Nat. Rev. Mol. Cell Biol. 16, 742–752 (2015).
12. Xu, C., Wang, S., Thibault, G. & Ng, D. T. W. Futile Protein Folding Cycles in the ER Are Terminated by the Unfolded Protein O-Mannosylation Pathway. Science (80-.). 340, 978–981 (2013).
13. Olzmann, J. A., Kopito, R. R. & Christianson, J. C. The mammalian endoplasmic reticulum-associated degradation system. Cold Spring Harb. Perspect. Biol. 5, (2013).
14. Ikezawa, H. Glycosylphosphatidylinositol (GPI)-anchored proteins. Biol. Pharm. Bull. 25,

409–17 (2002).
15. Fujita, M. & Kinoshita, T. Structural remodeling of GPI anchors during biosynthesis and after attachment to proteins. FEBS Lett. 584, 1670–1677 (2010).
16. Ron, D. & Harding, H. P. Protein-folding homeostasis in the endoplasmic reticulum and nutritional regulation. Cold Spring Harb. Perspect. Biol. 4, (2012).
17. Ron, E. et al. Bypass of glycan-dependent glycoprotein delivery to ERAD by up-regulated EDEM1. Mol. Biol. Cell 22, 3945–3954 (2011).
18. Mohorko, E., Glockshuber, R. & Aebi, M. Oligosaccharyltransferase: the central enzyme of N-linked protein glycosylation. J. Inherit. Metab. Dis. 34, 869–878 (2011).
19. Aebi, M., Bernasconi, R., Clerc, S. & Molinari, M. N-glycan structures: recognition and processing in the ER. Trends Biochem. Sci. 35, 74–82 (2010).
20. Aebi, M. N-linked protein glycosylation in the ER. Biochim. Biophys. Acta - Mol. Cell Res. 1833, 2430–2437 (2013).
21. Lord, C., Ferro-Novick, S. & Miller, E. A. The Highly Conserved COPII Coat Complex Sorts Cargo from the Endoplasmic Reticulum and Targets It to the Golgi. Cold Spring Harb. Perspect. Biol. 5, a013367–a013367 (2013).
22. Dancourt, J. & Barlowe, C. Protein Sorting Receptors in the Early Secretory Pathway. Annu. Rev. Biochem. 79, 777–802 (2010).
23. Bagola, K., Mehnert, M., Jarosch, E. & Sommer, T. Protein dislocation from the ER. Biochim. Biophys. Acta - Biomembr. 1808, 925–936 (2011).
24. Nakatsukasa, K., Brodsky, J. L. & Kamura, T. A stalled retrotranslocation complex reveals physical linkage between substrate recognition and proteasomal degradation during ER-associated degradation. Mol. Biol. Cell 24, 1765–75, S1-8 (2013).
25. Nakatsukasa, K., Kamura, T. & Brodsky, J. L. Recent technical developments in the study of ER-associated degradation. Curr. Opin. Cell Biol. 29, 82–91 (2014).
26. Hebert, D. N., Bernasconi, R. & Molinari, M. ERAD substrates: Which way out? Semin. Cell Dev. Biol. 21, 526–532 (2010).
27. Croset, A. et al. Differences in the glycosylation of recombinant proteins expressed in HEK and CHO cells. J. Biotechnol. 161, 336–348 (2012).
28. Moremen, K. W., Tiemeyer, M. & Nairn, A. V. Vertebrate protein glycosylation: diversity, synthesis and function. Nat. Rev. Mol. Cell Biol. 13, 448–462 (2012).

29. Stanley, P. Golgi glycosylation. Cold Spring Harb. Perspect. Biol. 3, (2011).
30. Bitsikas, V., Corrêa, I. R. & Nichols, B. J. Clathrin-independent pathways do not contribute significantly to endocytic flux. Elife 3, e03970 (2014).
31. Banfield, D. K. Mechanisms of Protein Retention in the Golgi. Cold Spring Harb. Perspect. Biol. 3, a005264–a005264 (2011).
32. Gannon, J., Bergeron, J. J. M. & Nilsson, T. Golgi and Related Vesicle Proteomics: Simplify to Identify. Cold Spring Harb. Perspect. Biol. 3, a005421–a005421 (2011).
33. Ispolatov, I. & Müsch, A. A model for the self-organization of vesicular flux and protein distributions in the Golgi apparatus. PLoS Comput. Biol. 9, e1003125 (2013).
34. Johnson, N., Powis, K. & High, S. Post-translational translocation into the endoplasmic reticulum. Biochim. Biophys. Acta - Mol. Cell Res. 1833, 2403–2409 (2013).
35. Lakkaraju, A. K. K. et al. Efficient secretion of small proteins in mammalian cells relies on Sec62-dependent posttranslational translocation. Mol. Biol. Cell 23, 2712–2722 (2012).
36. Vilardi, F., Lorenz, H. & Dobberstein, B. WRB is the receptor for TRC40/Asna1-mediated insertion of tail-anchored proteins into the ER membrane. J. Cell Sci. 124, 1301–1307 (2011).
37. Stefanovic, S. & Hegde, R. S. Identification of a Targeting Factor for Posttranslational Membrane Protein Insertion into the ER. Cell 128, 1147–1159 (2007).
38. Kallehauge, T. B. et al. Ribosome profiling-guided depletion of an mRNA increases cell growth rate and protein secretion. Sci. Rep. 7, 40388 (2017).
39. Martínez, V. S., Buchsteiner, M., Gray, P., Nielsen, L. K. & Quek, L.-E. Dynamic metabolic flux analysis using B-splines to study the effects of temperature shift on CHO cell metabolism. Metab. Eng. Commun. 2, 46–57 (2015).
40. van Wijk, X. M. et al. Whole-Genome Sequencing of Invasion-Resistant Cells Identifies Laminin α2 as a Host Factor for Bacterial Invasion. MBio 8, e02128-16 (2017).
