# Supplementary material for: Genome-scale reconstructions of the mammalian secretory pathway predict metabolic costs and limitations of protein secretion
Source: Nat Commun. 2020 Jan 2;11:68. doi: 10.1038/s41467-019-13867-y (PMC6940358; doi:10.1038/s41467-019-13867-y)
Supplement: Supplementary file 2 — Description of Additional Supplementary Files [file 41467_2019_13867_MOESM2_ESM.pdf]

Description of Additional Supplementary Files

File Name: Supplementary Data 1
Description: Reactions, components, and vesicle stoichiometry used in mammalian secretory pathway reconstructions

File Name: Supplementary Data 2
Description: Protein Specific Information Matrices (PSIM) of human, mouse, and Chinese hamster

File Name: Supplementary Data 3
Description: Experimental data and IgG amino acid compositions used to constrain iCHO2048s

File Name: Supplementary Data 4
Description: In silico assessment of CHO cell productivity improvements upon host gene deletions
